# Respiration shapes response speed and accuracy with a systematic time lag

**DOI:** 10.1098/rspb.2024.2566

**Published:** 2025-04-09

**Authors:** Cosima Harting, Lena Hehemann, Lisa Stetza, Christoph Kayser

**Affiliations:** ^1^ Biology, Bielefeld University, Bielefeld 33615, Germany

**Keywords:** respiration, perception, cognition

## Abstract

Sensory–cognitive functions are intertwined with physiological processes such as the heart beat or respiration. For example, we tend to align our respiratory cycle to expected events or actions. This happens during sports but also in computer-based tasks and systematically structures respiratory phase around relevant events. However, studies also show that trial-by-trial variations in respiratory phase shape brain activity and the speed or accuracy of individual responses. We show that both phenomena—the alignment of respiration to expected events and the explanatory power of the respiratory phase on behaviour—co-exist. In fact, both the average respiratory phase of an individual relative to the experimental trials and trial-to-trial variations in respiratory phase hold significant predictive power on behavioural performance, in particular for reaction times. This co-modulation of respiration and behaviour emerges regardless of whether an individual generally breathes faster or slower and is strongest for the respiratory phase about 2 s prior to participant’s responses. The persistence of these effects across 12 datasets with 277 participants performing sensory–cognitive tasks confirms the robustness of these results, and suggests a profound and time-lagged influence of structured respiration on sensory–motor responses.

## 1. Introduction

Recent studies highlight the relation between physiological processes such as respiration, heartbeat, gut activity and brain function [1–5]. Taking respiration as an example, we know that the brain structures controlling respiration and those sensing the resulting changes in airflow or chest pressure are intricately connected with the limbic nervous system [6–8]. As a result, direct neural feedback about the respiratory state is widely available in the brain [9–11]. Further, respiration-driven changes in blood oxygenation affect the brain’s metabolism and its supply with energy. Hence, respiration can both directly and indirectly shape how sensory information is encoded and translated into motor actions [12–14].

Several studies show that participants’ trial-to-trial performance in sensory–cognitive tasks covaries with the respiratory phase [12,13,15–20]. Still, it remains unclear whether this co-modulation relates to how well individuals perform a task, how fast they react (reaction times), or both. Reaction times may vary with respiration simply owing to changes in muscle tension or brain–muscle coordination with respiration [21–24]. By contrast, a covariation of response accuracy along the respiratory cycle may result from changes in neural excitability or inter-areal coupling [14,25], and hence point to a more profound modulation of sensory–cognitive processes. Furthermore, most studies focused on the immediate respiratory phase in each trial. However, given the multiple pathways by which respiration-related signals or changes in blood oxygenation can effect brain function, it remains unclear whether any co-modulation of respiration and behaviour is immediate or temporally delayed [6].

Humans tend to align their respiration to expected events or actions. For example, breath holding is used during shooting and fast motor actions are often accompanied by specific breathing patterns [26,27]. Similarly, the respiratory pattern of participants during neuro-cognitive laboratory tasks is structured and exhibits consistency across trials [28,29]. However, perfect consistency of the respiratory phase across repeats of the same action would leave little trial-to-trial variability in respiration to explain variability in the action outcome. We here investigate how both the systematic alignment of respiration to task-events and trial-to-trial variations in respiratory phase that are predictive of behaviour can co-exist.

We leverage a large dataset to systematically probe the alignment of respiration to experimental trials and the relation between task performance (response accuracy, reaction times) and the respiratory phase seconds before each trial. This dataset comprises 12 experiments implementing sensory and cognitive tasks. Participants performed these tasks without a specific constraint on how to breathe. This instruction was used to mimic typical ‘every-day’ experiments that do not impose specific manipulations on participants' respiration. The timing of individual trials followed a typical rapid and pseudo-random sequence of events in which trials are spaced a few seconds apart and preceded by a baseline period that cues participants' attention to the upcoming stimulus. The results show that participants tend to align their respiration to individual trials, resulting in a consistent pattern of respiration both across trials for individual participants and on average across trials. At the same time, behavioural performance is co-modulated with the residual variability of the respiratory phase. This co-modulation is strongest when measured against the respiratory phase about 2 s prior to the individual responses, suggesting a profound and time-lagged influence of respiration on performance in typical sensory–cognitive laboratory tasks.

## 2. Methods

### (a) Participants

All studies were approved by the ethics committee of Bielefeld University. Adult volunteers participated after providing informed consent and were compensated for their time. All had self-reported normal vision and hearing. The data were collected anonymously and it is possible that some individuals participated in more than one of the experiments described below. Demographical data were not collected, but we expect these to be very similar to previous studies, in particular as the participant pool consisted of typical young university students [30,31]. The specific interest in investigating the relation between respiration and task performance was not mentioned explicitly prior to the study. Participants were instructed to ‘breathe through their nose as usual’, as if performing the experiments without wearing the mask. During the experiment we could not continuously monitor whether participants adhered to this instruction, leaving the possibility that during parts of the experiment participants were breathing orally.

### (b) General procedures

The experiments were performed in a darkened and sound-proof booth (E: Box; Desone, Germany). In one line of experiments, visual stimuli were presented on a computer monitor (27’ monitor; ASUS PG279Q, about 1 m from participant’s head) while acoustic stimuli were presented from two speakers placed besides the monitor. In another line of experiments, participants sat in front of an acoustically transparent screen (Screen International Modigliani, 2 × 1 m) onto which visual stimuli were projected (LG HF65, LG electronics) while acoustic stimuli were presented from headphones (Sennheiser DH200Pro). Acoustic stimuli were calibrated to have an r.m.s. level of about 65 dB SPL. Stimulus presentation was controlled using the Psychophysics Toolbox (v. 3.0.14) using MATLAB (v. R2017a; The MathWorks, Inc., Natick, MA) and was synchronized to a BioSemi EEG recording system using TTL pulses. Participants responded using computer keyboards.

### (c) Behavioural paradigms

We analysed both data from experiments collected *de novo* (datasets 1−7) and from experiments performed and published in a previous study (datasets 8−12) [28]. All experiments involved one or more blocks of experimental trials, a practice block and possibly a block to determine each participant’s perceptual threshold for the respective task. For each task, participants were instructed to respond as fast and accurately as possible after stimulus presentation.

**Figure 1.**
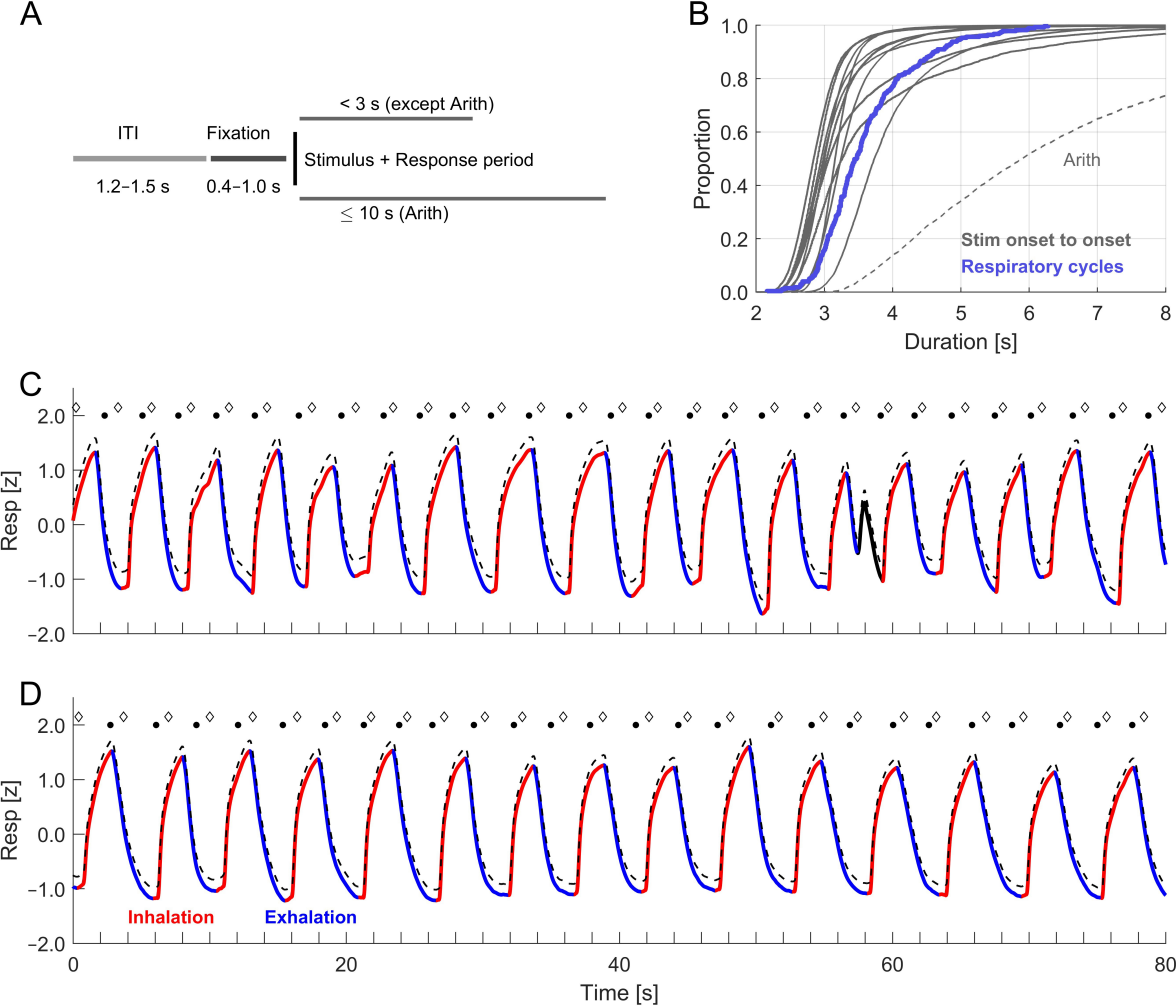
Methodology. (A) Schematic of the experimental design that was similar for most paradigms. All experiments consisted of sequences of trials, which were separate by random inter-trial intervals of 1.2−1.5 s duration. Stimulus onset was cued by a fixation period of random duration (0.4–1.0 s uniform). In most paradigms participants could respond immediately after stimulus onset. The response period was constrained to less than 3 s for all paradigms except Arithmetics, for which participants were given 10 s to respond. (B) Cumulative distribution of the actual intervals between subsequent stimulus onsets for each paradigm (grey lines). For all paradigms except Arithmetic these were on the order of 3 to 4 s, which is the same time scale as the duration of typical respiratory cycles (blue distribution, pooled across all participants). (C-D) Example data for the respiratory signal for two participants. The dashed black line is the original signal from the temperature probe and the solid line is the processed and filtered signal, based on which the respiratory cycle was split into inhalation and exhalation periods. In panel (C), the solid black part of the signal trace reflects a brief portion of the respiratory signal that was not classified and omitted from analysis. Black dots and diamonds indicate the onsets of the fixation period and the stimulus respectively for individual trials.

The design of all experiments was similar (figure 1A). Trials started with a fixation period indicated by the appearance of a central fixation dot that remained on the screen until the stimulus (400−1000 ms duration, drawn from a uniform distribution), followed by stimulus presentation, the duration of which differed between experiments. Participants could respond at any time after stimulus onset (except ‘Time perception’). The individual trials were separated by inter-trial intervals, drawn from a uniform distribution between 1200 and 1500 ms; these started once a response was submitted and lasted until the onset of the fixation period of the next trial. The distribution of intervals separating subsequent stimulus onsets is shown in figure 1B. The typical pacing of trials was on the order of around 3−4 s, as typical for behavioural and neuroimaging paradigms probing sensory or cognitive processes. Only for the paradigm probing arithmetic was this duration longer, as during this paradigm the participants were given more time to respond (see below).

#### (i) Datasets collected in this study

*Pitch discrimination 1*: Participants compared the pitch of two brief successive tones, similar to [32]. During each trial two tones (50 ms duration, 6 ms cosine ramp, 50 ms pause in between, 65 dB SPL) were presented and participants indicated which of the two had higher pitch. Pitch differences were titrated around participants' thresholds in five levels, with a total of 600 trials (*n* = 16 participants).

*Time perception*: Participants categorized the duration of acoustically presented intervals as either ‘short’ or ‘long’. Intervals lasted 200, 250, 300, 350 or 400 ms, with the 300 ms interval being ambiguous and not analysed. Participants could only respond after stimulus offset. Stimuli were 1024 Hz tones and participants performed 540 trials (*n* = 18).

*Emotion discrimination 1*: Participants discriminated emotional expression (sad or happy) in briefly presented faces (subtending 15 × 12 degrees, presented for approx. 17 ms). Stimuli were obtained from the Dynamic FACES database [33]. For 24 individual faces, we selected images that either reflect each emotion clearly, each emotion to a mild degree or were neutral. Emotions were expressed in five levels, with the intermediate level being emotionally neutral and not analysed. Each participant completed 480 trials (*n* = 25).

*Visual shape discrimination 1*: Participants discriminated the orientation of briefly presented Necker cubes (subtending 11 × 9 degrees, presented for approx. 50 ms). For each cube four edges had an increased luminance to render the orientation of the shape less ambiguous, with a total of five levels (the intermediate level being ambiguous and not analysed). Each participant completed 480 trials. Inter-trial intervals were 800−1200 ms (uniform) (*n* = 25).

*Pitch discrimination 2*: This was the same as pitch discrimination 1, except that there were only two levels of pitch difference. Prior to each block of this task participants performed 1 min of structured respiration training (either box-breathing or hyperventilation). For the present study, we ignored the training condition. 480 trials per participant (*n* = 52).

*Emotion discrimination 2*: Participants discriminated the emotion (anger or disgust) in briefly presented faces (subtending 10 × 10 degrees, presented for 128 ms). Images were taken from the FACES database [34]. A total of 100 images were used and each participant completed 400 trials. Prior to each block participants performed 1 min of structured respiration training (either box-breathing or hyperventilation) and we obtained 523 trials per participant (*n* = 24).

*Arithmetic*: Participants performed arithmetical tasks requiring the summation of either a one- and a two-digit number (easy trials) for two two-digit numbers (difficult trials). These numbers were presented on the screen and participants were given a 10 s to enter the respective sum. Note that this response period is much longer than the typical response periods during the other paradigms (figure 1B). Participants performed a total of 270 trials, of which 135 were considered easy and 135 as difficult. Prior to each block participants performed one minute of structured respiration training or breathed normally (*n* = 18).

#### (ii) Previously published datasets

These have been described in detail in [28]. From this previous publication we re-analysed all five sensory tasks (ignoring the memory paradigm also reported there) using revised procedures. These include visual motion discrimination (*n* = 18), pitch discrimination 3 (identical to pitch 1, *n* = 20), pitch discrimination 4 (here participants had to manually initialize the start of the trial by pressing a key; *n* = 20), sound detection (*n* = 20) and emotion discrimination (*n* = 21).

### (d) Recording of respiratory signals

Respiration was recorded using a temperature-sensitive resistor that was inserted into disposable clinical masks (Littelfuse Thermistor No. GT102B1K, Mouser electronics). This captures the continuous temperature changes resulting from the respiration-related airflow [28,35]. The voltage drop across the thermistor was recorded via an ActiveTwo EEG system (BioSemi BV) at a sampling rate of 500 or 1000 Hz. We verified that the voltage drop of the temperature sensor follows the respiratory airflow without time lag. For this we combined the temperature probe with two short-latency airflow sensors (F1031V, Mass Airflow Sensor, Winsen) and confirmed that the temperature change tightly aligns with the directional change in airflow. Example signals are shown in figure 1C,D.

### (e) Analysis of respiratory data

The respiratory signals were filtered using third-order Butterworth filters (high pass at 0.03 Hz, low pass at 6 Hz) and subsequently resampled at 100 Hz using the FieldTrip toolbox [36]. The signals were then converted to *z*-scores to facilitate comparison across participants (figure 1C,D). To detect individual respiratory cycles, we applied the Hilbert transform to determine local peaks based on the respective phase [37]. Individual respiratory cycles were determined based on the data in windows of 7 s around each peak, whereby individual peaks were only retained for further analysis if the *z*-scored trace exceeded a value of *z* = 0.5 [13]. Note that alternative algorithms to detect individual respiratory cycles exist and in a previous study we found little difference between these [28]. The inhalation period was defined as the continuous period with positive slope prior to the local peak (whereby interruptions of the positive slope shorter than 500 ms were interpolated). The exhalation period was defined as the continuous period with negative slope subsequent to the local peak (again, interruptions shorter than 500 ms were interpolated). This definition effectively splits the respiratory cycle effectively into inhalation and exhalation. although for some cycles short exhale pauses were classified as third state and not analysed [37]. To characterize atypical respiratory cycles, we compared the overall time courses of individual cycles using their mean-squared distances. We calculated the participant-wise distributions and excluded cycles with a distance larger than 3 standard deviations (SDs) from the centroid. Further individual trials were excluded from analysis as noted below. From the full datasets we retained only participants for whom these procedures excluded less than 30% of the available trials for the final statistical analysis.

**Figure 2.**
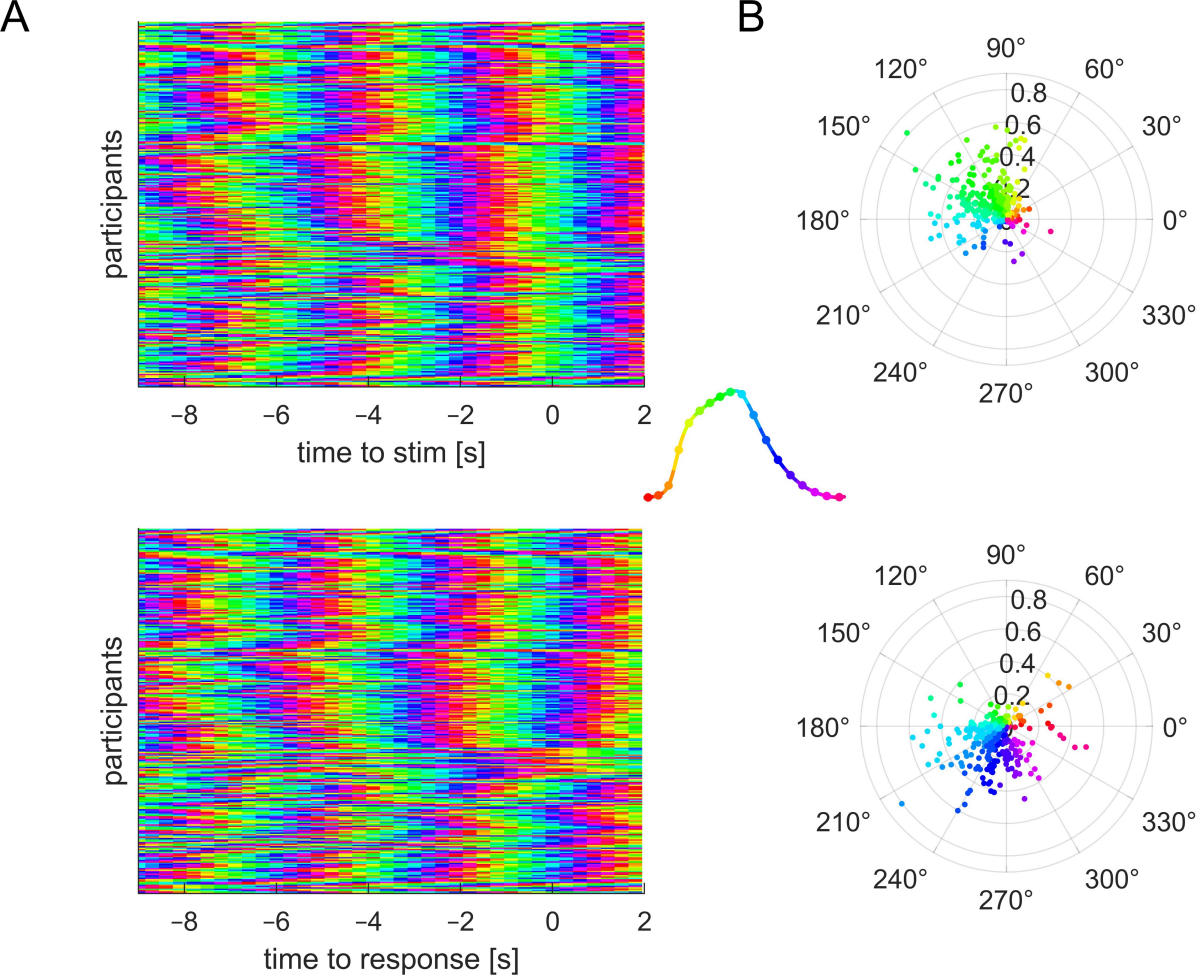
Trial-averaged respiratory phase for each participant. (A) Average phase for the respiratory phase derived relative to stimulus onset (top) or response times (bottom). Note how the phase becomes more similar across participants around each trial (near time zero). (B) Polar plot of the participant-wise average phase together with the participant-wise phase-locking value (coded as distance from the centre). Note how the population shifts from a greenish phase to blueish phase from stimulus onset to the response. The inset in the middle shows the colour-coding of phase along a prototypical respiratory cycle (inhalation upwards).

To link respiratory signals to behaviour, we defined the phase of each respiratory cycle as a linearly increasing variable from the beginning to the end of inhalation (defined as angle from 0 to π) and subsequently as linearly increasing from the beginning to the end of exhalation (defined as π to 2×π). This phase variable scales linearly in time within each inhalation or exhalation period and has three time points (0, π, 2π) whose interpretation is consistent regardless of the shape of individual respiratory cycles. Figure 2 indicates this phase variable for one prototypical respiratory cycle.

### (f) Statistical analysis of the alignment between respiration and paradigm

**Figure 3.**
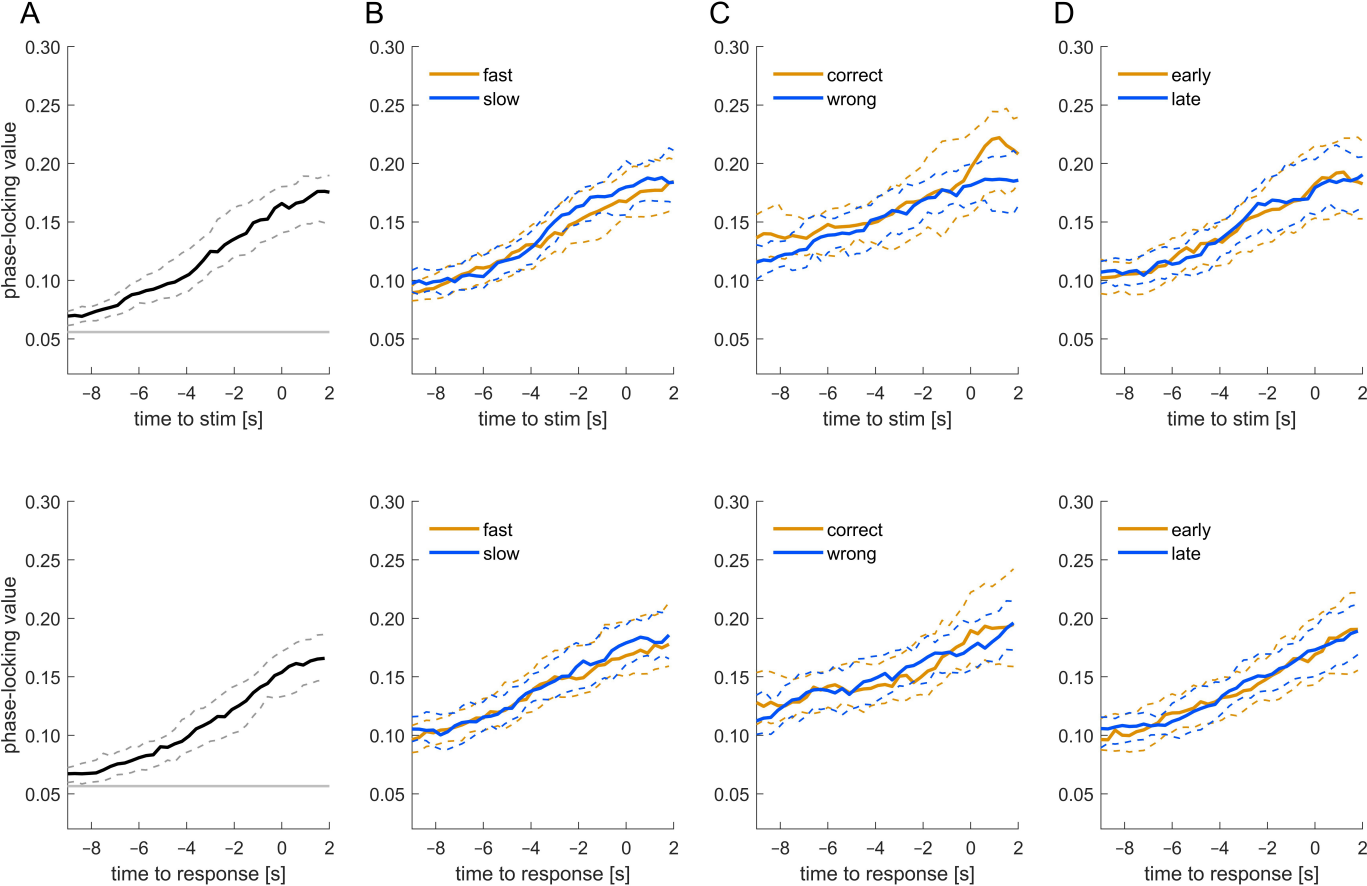
Consistency of respiratory phase across trials within participants. The figure shows the group-level phase locking values for the data aligned to stimulus onset (upper panels) or participants’ responses (lower panels). The four panels show the phase-locking computed across all trials (A), trials split by the participant-wise median reaction times (B), response accuracy (C) and the time-on-experiment (D). Solid lines indicate the group median, dashed lines the 95% bootstrap confidence interval. The grey horizontal line in panel (A) shows the value corresponding to a significance of *p* < 0.001 (randomization test; corrected for multiple tests across time points). The sample size is 277 for all panels except (C), where some participants had to be excluded because they featured too few trials to estimate phase-locking separately for correct and wrong trials (*n* = 208).

To probe whether and how participants aligned their respiratory behaviour to the experimental trials we computed the phase consistency (phase-locking) across trials (figure 3). This was done by converting the phase into a complex-valued number, averaging this across trials and taking the vector length. To test for the statistical significance of this, we derived a surrogate distribution of phase-locking values under the null hypothesis of no alignment between respiratory trace and paradigm for each participant. This was obtained by randomly time-shifting the respiratory trace and recalculating the phase consistency 4000 times. We then compared the actual group-median distribution of group-medians in the surrogate data, taking the maximal value along the time axis to correct for multiple comparisons.

Both the respiratory cycle and the timing of subsequent trials exhibit a pseudo-periodic structure, the latter because typical inter-trial intervals were random only within a certain time window (c.f. figure 1B). As a result, a strong and mechanistic phase-locking of both signals at one time point (e.g. stimulus onset, or response times) would inevitably also result in phase locking at other time points. For this reason, we do not interpret the precise time window within which phase-locking is significant (or not significant), but rather emphasize that phase-locking is significant (versus a suitable baseline) and the time points at which this is strongest.

We also tested whether this alignment of respiration to the experimental trials differed between trials with fast/slow responses, correct/wrong responses and trials in the first/second half of each experiment (figure 3). For reaction times we relied on a median split of trials, while for time-on-the-experiment we split trials based on the actual trial numbers. For response accuracy the number of correct and wrong trials was highly imbalanced. Since this may bias the phase-locking estimate, we used equal trial numbers for each group and excluded participants for whom fewer than 40 trials were available per group.

### (g) Analysis of the co-modulation of behaviour with respiration

For this we relied on linear mixed effect models (equation (2.1)). Separate models were fit for reaction times and accuracy, for each of the datasets, and separately for the phase of respiration at different time points relative to the experimental paradigm. As predictors we included a variable reflecting a parametric manipulation of task difficulty (e.g. the pitch difference; termed *Stimlevel* in equation (2.1)), or for those tasks without such a parametric variable, one reflecting the two conditions to be discriminated. We also included a participant-wise (*Sid*) random effect of trial number (*TrialNr*) to capture potential effects of fatigue or training-on-the-task and a random offset for each participant. Respiration was modelled using the sine- and cosine-transformed trial-wise phase of respiration as separate predictors, at particular time points of interest. These time points were either the time of stimulus onset, the time at which participants responded in each trial, or a time point at a particular lag preceding either stimulus onset or responses, defined in steps of 300 ms. We did not use a finer spacing of time points given the high redundancy of the respiratory phase among neighbouring time points (300 ms correspond to about 1/12^th^ of the average respiratory cycle). Separate models were fit for the respiratory predictors extracted from individual time points. Note that this model implies a consistent relation between behaviour and respiratory phase across participants, if such an effect exists.


(2.1) RT∼Stimlevel+RespSine+RespCosine+(TrialNr|Sid)+(1|Sid)

Reaction times were square-root transformed before entering the model. Models for reaction times used a linear activation function and assumed Gaussian variables; models for accuracy assumed binomial variables and a logistic activation function.

### (h) Statistical analysis

We derived two measures of statistical significance for an effect of respiration in these models. The first focused on the predictive power of respiration to explain trial-to-trial variability in behaviour. For this we compared the Akaike information criteria (AIC) between two models, of which only one included respiration as factor (that is, the other model omitted both the sine and cosine predictors). Based on this we derived the conditional probability of each model given the data, i.e. the associated Akaike weights [38]. We considered an AIC difference > 9.2 as evidence for an effect of respiration, which corresponds to a probability of above 99% that the model including respiration has more explanatory power than the alternative model of above 99%.

As a second measure of significance we focused on the slope of the respiratory predictors. For this we compared the vector strength of the combined sine and cosine predictors in the actual data with those in surrogate data [10,28]. The vector strength was defined as the geometric average of the respective sine and cosine terms. To obtain surrogate data we shuffled the trial-wise relation of the dependent and independent variables and computed the models again, repeating this 4000 times. We then derived the *p*-value of the vector strength against the surrogate data for each experiment. The resulting 12 *p*-values were combined across datasets using Fisher’s procedure [39]. Given the computational burden arising from this procedure, we implemented this using the respiratory phase at two time points (−2.1 s, 0 s) relative to the stimulus or response (electronic supplementary material, table S1).

Using similar model comparison, we tested for an effect of the stimulus level or condition. This corresponded to an AIC weight corresponding to an AIC weight of above 99% for 10 (of 12) datasets for accuracy and for 10 (of 12) datasets for reaction times, showing that the stimulus manipulations were predictive of behaviour.

For these analyses several trials had to be excluded for the following reasons: either the phase of respiration was not defined at the time point of interest, or the reaction time was deemed an outlier. The latter were defined as overly short (<200 ms) or excessively long reaction times (>3 s). Finally, a few trials had to be removed because participants pressed a wrong button. Collectively this led to the exclusion of 8.6 ± 3.4% (mean ± SD) of the available trials. Overall, we included 107 432 trials from 277 participants in the analysis.

**Figure 4.**
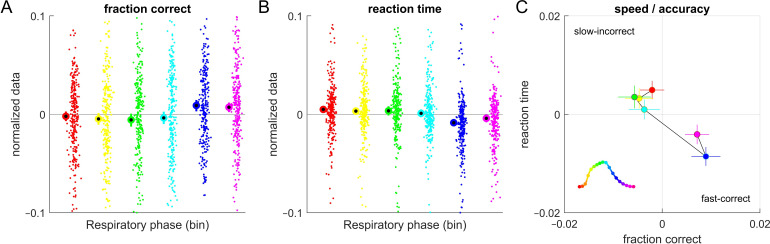
Behavioural data versus respiratory phase. (A) Fraction of correct responses for trials featuring either of six (binned) respiratory phase angles. Dots indicate individual participants, large circles show the bin-average. The data were mean-normalized for each participant for better visualization. (B) Reaction times for each respiration phase bin. The single trial reaction times were square-root transformed and mean-normalized for each participant for visualization. (C) Group-level mean and s.e.m. of fraction of correct responses and reaction times for each phase bin. The respiratory phase was derived 2.1 s prior to the response (corresponding to the peak in figure 5B). The inset shows the colour-coding of phase against a prototypical respiratory trace.

To visualize the behavioural data against the trial-wise respiratory phase (figure 4) we partitioned the phase angles into six equally-spaced bins and computed for each participant the fraction of correct responses and the median reaction time (based on the square-root transformed data, as used for linear models). To emphasize variability over phase bins rather than participants we subtracted the bin-averaged performance (reaction time) for each participant.

**Figure 5.**
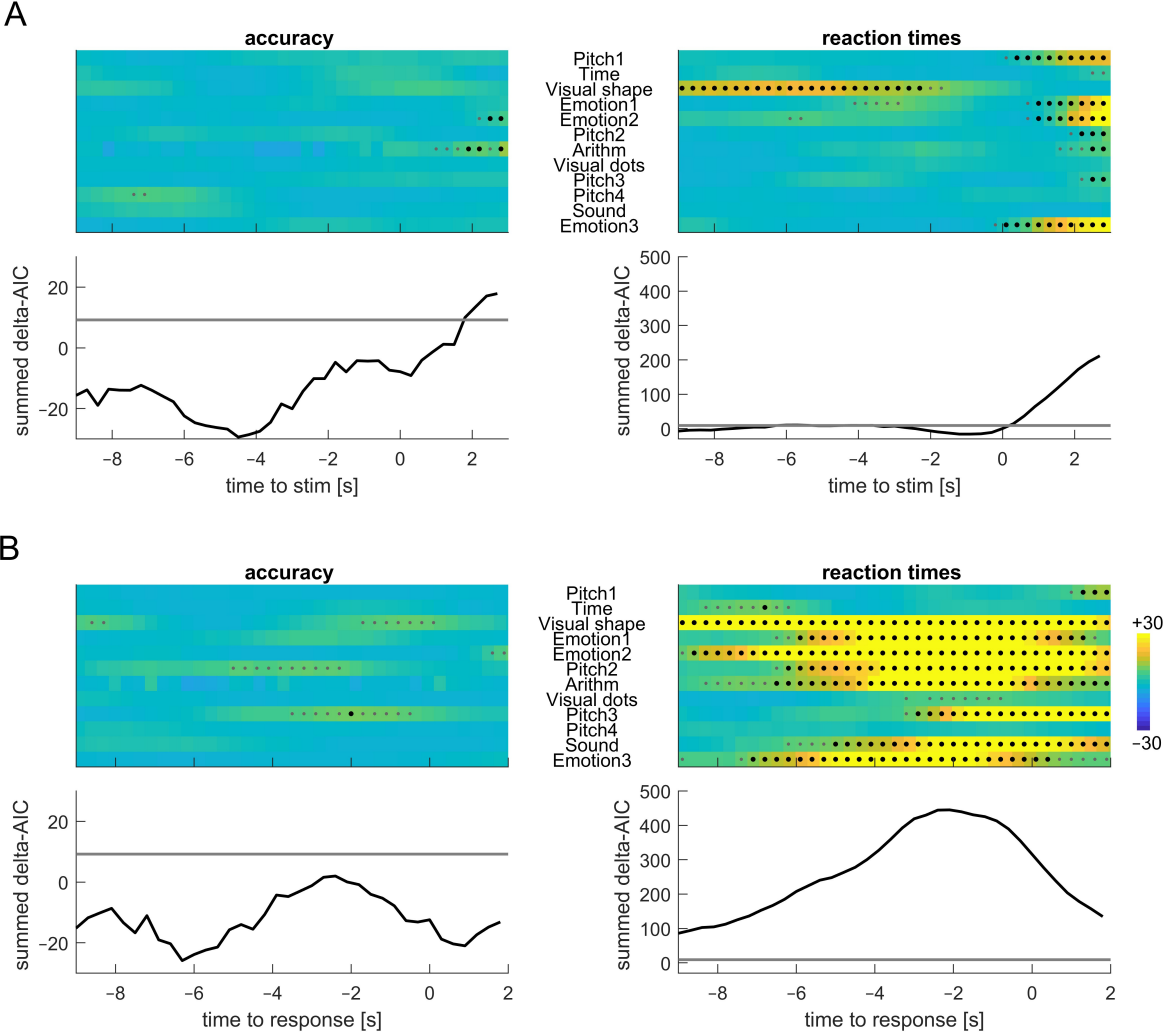
Co-modulation of behaviour and respiration. The statistical evidence in favour of a relation between respiration and trial-wise response accuracy or reaction times is shown as the AIC difference between a model including respiration and a model not including this as a factor. Positive numbers indicate evidence in favour of an effect. Part (A) shows the results for respiration aligned to stimulus onset. ‘part (B) shows the same for data aligned to response times. The colour-coded data show the outcome for each of the datasets, the graphs the summed AIC difference across all datasets. The grey (black) dots indicate AIC differences corresponding to an AIC weight of 95% (99%) in favour of an effect of respiration.

To relate the trial-average respiratory phase to behaviour (electronic supplementary material, figure S1), we implemented the following in-between participant analysis. We derived for each participant the trial-averaged phase at −2.1 s prior to the response, and divided these phase values into the six equally spaced phase bins. We then visualized the participant-wise fraction of correct responses (across all trials) and the median reaction time (across all trials) for participants in each bin. To avoid outliers, we only included participants whose median reaction time was below 2 s. Because many participants shared a similar average phase, the effective sample size per phase bin differs.

### (i) Analysis split by respiration rate

We split participants into those breathing faster and those breathing slower using a median split on the average duration of the respiratory cycle. Because the numbers of fast and slow breathers differed between datasets, we probed the predictive power of respiration on behaviour for each group as follows: we derived the group-level AIC values by fitting linear models to the individual participant data (similar to equation (2.1), but without the participant ID as random effect). The individual AIC values were then summed across participants per group (electronic supplementary material, figure S2B).

To visualize the respiratory time course for fast and slow breathers separately (electronic supplementary material, figure S2A), we first determined for each group a prototypical respiration curve. This was obtained by finding those 10 participants whose inhalation and exhalation durations were closest to the average of the respective groups and computing the trial-averaged shape of one respiration cycle for these participants. This cycle was then appended a few times to cover the time window to be displayed. This prototypical curve was then shifted so that the phase at the time of response reflects the group-average phase at this time. Note that this implicitly assumes that subsequent cycles are identical and is mainly intended for illustration.

## 3. Results

The average duration of respiratory cycles was 3.6 ± 0.7 s (mean ± SD) with exhalation periods being longer (1.9 ± 0.4 s) than inhalation periods (1.6 ± 0.4 s). Figure 1B shows the cumulative distribution of respiratory cycles across all participants.

### (a) Respiration aligns to experimental trials

To confirm that respiration was systematically structured relative to the experiments, we measured the between-trial consistency of respiratory phase. Figure 3 shows the group-level phase-locking index for all trials, trials split by reaction time, accuracy or time-on-the-experiment; the data are also shown separately for respiration relative to stimulus onset (upper panels) and the times when participants responded (lower panels). Phase-locking was stronger than expected by chance (randomization tests, *p* < 0.001; grey line in figure 2A) and was systematically higher around the stimulus/response period (near time 0 s) compared with a few seconds earlier. We did not observe significant differences in phase-locking when splitting trials by accuracy, reaction times or time-on-the-experiment (Wilcoxon sign-rank tests, at time points *t* = 0 s, all *p* > 0.54, Z < 0.61). Hence, participants' respiratory cycles are significantly structured relative to the sequence of experimental trials, without clear differences between trials characterized by the just-mentioned properties.

Figure 3A shows the trial-averaged respiratory phase for each participant, showing that the trial-averaged phase is also similar across participants. Panel (B) illustrates the average phase together with the phase-locking for each participant. Phase-locking is significantly stronger for respiration aligned to stimulus onset compared with response times, although the numeric differences are small (median values 0.191 versus 0.184; Wilcoxon signed rank test, *p* < 0.0001, *Z* = 3.9). The between-participant consistency of the participant-wise phase angles (also measured using phase-locking) did not differ significantly between stimulus- and response-aligned data (0.142 versus 0.108; randomization test, *p* = 0.14). This suggests that participants' respiration is systematically organized around the individual trials both within and between participants.

### (b) Behaviour is modulated along the respiratory cycle

We tested whether the respiratory phase has predictive power on the trial-wise response accuracy or reaction times using principles of model comparison. Figure 5 shows the AIC differences for an effect of respiratory phase tested at different time points prior to stimulus onset or response times (in 300 ms steps). The strongest evidence for a co-modulation of behaviour with respiration was observed for response-aligned data. Here, the evidence for a co-modulation of reaction times with respiration was very strong and the AIC weight (probability that the model including respiration fits the data better) was above 99.999%. For 10 (of 12) datasets we obtained an AIC weight corresponding to at least 99% in favour of an effect of respiration (all except Pitch 4 and Visual dots; indicated by black dots in figure 5). For response accuracy only five datasets featured an AIC weight corresponding to a probability of above 95% in either alignment of the data (these were Visual shape, Emotion 2, Arithm, Pitch 2 and Pitch 3). At the group level, the AIC difference was in favour of no effect of respiration. Importantly, the present data suggest that the peak relation between respiration and behaviour emerges around 2 s prior to a participant’s responses. To confirm this, we compared the predictive power of a model relying on the respiratory phase at 2.1 s prior to participants’ response (peak time) with one relying on the phase directly at the response time (*t* = 0 s): this provided clear evidence for respiration around *t* = −2.1 s being the better predictor (delta AIC = 129, AIC weight of above 99.999%).

Figure 4 illustrates this co-modulation by binning the trial-wise respiratory phase into six bins (defined 2.1 s prior to the response); for each bin we calculated the average reaction time and the fraction of correct responses. The fraction of correct responses is highest around the blue phase, where reaction times are shortest, while the opposite is observed near the yellow/red phase.

As a further test of the predictive power of respiration on behaviour, we probed whether vector-length of the respiratory predictors for the actual data was stronger compared with surrogate data. This effectively constitutes a different statistical test of the main question, relying on the slope of the respiratory predictors themselves rather than on metrics for model comparison. The results (electronic supplementary material, table S1) corroborate that the respiratory predictors are highly significant, in particular for reaction times and the response-aligned respiratory phase.

Given the typical reaction times in these datasets and the timing of experimental trials, the time point *t* = −2.1 s prior to each response could theoretically fall anywhere between the current stimulus presentation or the previous trial’s stimulus. We directly calculated for each paradigm and trial where this time point fell in each trial (except for Arithmetic). This revealed that for about 7% of trials this time point (*t* = −2.1 s prior to each response) was in the response period of the same trial, for 13% it was in the fixation period of the current trial, for 75% in the previous inter-trial interval and for only 5% of trials it fell into the stimulus/response period of the previous trial.

### (c) No influence of overall respiration rate

Given the inter-individual differences in average respiration rate (cycle durations: median 3.5 s, range 2.1 s and 6.3 s; figure 1B), it is possible that the statistical co-modulation of behaviour and respiration is expressed differently for participants breathing faster and slower. For example, the duration of the individual respiratory cycle constrains how strongly respiration can be aligned to the sequence of individual trials, as longer respiratory cycles are more difficult to align to individual trials on the time scale of 3 to 4 s. Indeed, the average respiratory rate was significantly anticorrelated with the phase-locking value (Spearman rank correlation, *r* = −0.57, *p* < 10^–10^, 99% confidence interval [−0.69 −0.43]). As a result, the individual respiratory rate may affect how well between-trial variations in respiratory phase can explain between-trial variations in behaviour. To investigate this, we split the participants into those breathing fast or more slowly (median cycle durations 3.1 and 3.9 s, respectively).

Electronic supplementary material, figure S2A visualizes exemplary respiratory traces for each group. This reveals that both groups exhibit a similar average respiratory phase around the response times. We confirmed that for both groups there is indeed a significant co-modulation of the trial-to-trial fluctuation in reaction times and respiratory phase (electronic supplementary material, figure S2B; peak delta-AIC values: 229 and 898; corresponding to a model probability of above 99.999%). Electronic supplementary material, figure S2C shows the behavioural data for individual respiratory phase bins, illustrating that for both groups the reaction times are fastest around the same phase. Still, the co-modulation was stronger for the fast breathers, as reflected by the differences in reaction times and accuracy between phase bins (electronic supplementary material, figure S2C): for example, for fast breathers the difference in accuracy between the most-extreme respiratory phases was twice as large compared with the slow breathers (0.026 versus 0.012).

### (d) Average respiratory phase is also predictive of behaviour

The above results show that participants tend to align their average respiratory phase to the experimental trials, but also that trial-by-trial variations in respiratory phase are predictive of behaviour. Together this suggests that participants who tend to align their respiration with the ‘optimal’ phase may on average perform better (or faster) than participants whose average phase is non-optimal. To test this, electronic supplementary material, figure S1 shows the behavioural data for the participant sample split by the trial-average respiratory phase of each participant (defined at *t* = −2.1 s). While this reveals great heterogeneity across participants, it also suggests a difference in reaction times between those individuals aligning with an exhalation phase (cyan/blue bins) and those aligning with onset of inhalation (red/yellow bins). Because the sample size differs per bin, we restricted the statistical testing to the comparison of reaction times between the combined red/yellow bins (*n* = 94 participants) versus those in the cyan/blue bins (*n* = 66 participants): this revealed significant differences for reaction times but not the fraction of correct responses (two-sample *t*-tests; reaction time: *t* = 4.90, *p* < 10^–5^; correct responses: *t* = −0.21, *p* = 0.83).

## 4. Discussion

Participants tend to align their respiration to the expected timing of a sequence of experimental trials. At the same time, the respiratory phase holds significant predictive power on behavioural performance both across trials for an individual and across the sample of participants. This relation of respiration and behaviour emerges regardless of whether an individual breathes faster or slower and is strongest when considering the respiratory phase around 2 s prior to the individual responses. Our results demonstrate these results across a large sample of data from different typical sensory–cognitive tasks, confirming the ubiquity of this relation between respiration and behaviour.

### (a) Respiratory phase displays consistency and variability relative to experiment trials

It is known that humans tend to breathe in a structured manner around expected events, such as during sports, during conversation and also in a laboratory task [26,28,29,40,41]. This alignment of respiration to such events manifests is a consistency of the respiratory phase across trials. One factor driving this alignment is the partial predictability of typical experimental trials, as each upcoming stimulus is cued by a fixation period and both the inter-trial intervals and the duration of these fixation periods are randomized only within a restricted range of intervals (c.f. figure 1). This allows, to some degree, the prediction of the upcoming stimulus, the timing of which is on the same order as the duration of typical respiratory cycles. Furthermore, participants know when they will respond, and hence can also time their respiration relative to their motor actions.

This phase-locking was comparable for stimulus- and response-aligned data. This is not surprising given that for most paradigms the duration of stimulus presentation was short and reaction times were between 0.51 and 1.36 s (the time between stimulus onset and response; 10^th^ and 90^th^ percentile across all trials). This makes a differential alignment of respiration to stimulus onset and response times practically difficult, given that the typical respiratory cycle duration was around 3.4 s (overall median). In a previous study, we concluded that respiration was significantly more strongly phase-locked to the response [28]; however, the numerical differences were also small. Given the slow duration of individual respiratory cycles compared with the faster sequence of experiment trials, it is inherently difficult to determine statistically whether respiration was aligned actively and selectively to either an expected stimulus or to the planned motor actions. Owing to this temporal autocorrelation of the respiratory signal and the overlapping time scales of respiration and the timing of experimental trials (c.f. figure 1B), the phase-locking of both is visible over an extensive time period. Consistently across studies, however, the average tendency of the respiratory phase is to inhale around stimulus presentation and to exhale around responses.

Besides this alignment, the individual respiratory phase is predictive of behaviour. This was visible for individual participants in the trial-to-trial data and was stronger for the respiratory phase measured relative to the response time rather than to the stimulus onset. This implies that the trial-to-trial variability of the difference in respiratory phase between stimulus onset and response time is sufficient to account for their difference in predictive power against behaviour. Indeed, the overall median reaction time (0.73 s) corresponds to half the median duration of an inhalation period (1.48 s), which is sufficient to shift the respiratory phase from a phase angle related to faster response to one related to slower responses (c.f. figure 4). Furthermore, the trial-averaged respiratory phase is also predictive of between-participant variations in reaction times: participants who align ‘optimally’ to the paradigm tended to respond faster than those who aligned ‘non-optimally’. Hence, respiratory phase has a profound and systematic relation to the speed of responses.

### (b) Time-lagged relation of respiration to behaviour

The predictive power of respiration on behaviour peaks about 2 s prior to the response, independently of how fast an individual breathes. This time lag provides clues about the putative mechanisms underlying the co-modulation of respiration and behaviour. A short-latency co-modulation may be explained by direct neural feedback about respiratory movements or resulting airflow [6–9]. By contrast, a time-lagged effect leaves the possibility that respiration-related changes in heart rate or blood oxygenation contribute. While it takes seconds for oxygen to reach the brain from the lungs, the time scale of 2 s is also sufficient to detect changes in the instantaneous heart rate. The heart rate and respiratory phase are directly and indirectly coupled e.g. by common inputs from the autonomic nervous system [42,43]. Information about the timing of the heart beat is relayed by the vagus nerve and is available to interoceptive brain regions [44–47]. Similar to respiration, studies show that the relative timing of stimuli to the heartbeat shapes how they are perceived or memorized [48–51]. Furthermore, respiration is known to modulate cortical signatures of neural excitability and arousal [14,25], possibly by modulating activity of the locus coeruleus [6,52]. To better understand the pathways by which a time-lagged influence of respiration on sensory–cognitive processes and motor actions emerges, future work needs to dissociate the multiple bodily and neuro-physiological signals and their relation to behaviour [4].

### (c) Does respiration relate to both reaction times and response accuracy?

Previous work suggests that respiration may shape both reaction times and response accuracy. As we recently demonstrated [28], changes in response accuracy along the respiratory cycle are typically between 1% and 5%, and many studies failed to find significant changes in accuracy. By contrast, changes in reaction times are reported more frequently and the effect sizes are comparable with changes induced by brain stimulation or classical cognitive interference effects [28]. Here, the evidence for a relation between respiration and behaviour was stronger for reaction times compared with accuracy. Only for 5 out of 12 datasets did we find a significant predictive power of respiration on response correctness.

While this may suggest that response speed is generally more influenced by respiration, one cannot rule out that differences in the statistical sensitivity contribute to this result. Given the continuous and discrete nature of the trial-wise reaction times and accuracy values, we relied on different statistical models for these data; this may at least theoretically result in a differential sensitivity. Indeed, the group-level averages reveal a visible modulation in both aspects of behaviour. However, in a previous study we relied on a different statistical approach (see also below), which did not require different models for reaction times and accuracy [28]. Still, we found more prominent effects for reaction times. All in all, this calls for future studies to more finely dissociate effects of respiration on the quality of sensory representations that may shape response accuracy and more peripheral effects such as muscle tone or brain–muscle communication [14,24].

Future work could also investigate in more detail to what degree this co-modulation of behaviour with the respiratory cycle depends on the mode of breathing. Some previous studies reported stronger effects for nasal versus oral breathing [12,13] suggesting that the sensation of airflow by neurons in the nose as well as potential olfactory cues may contribute independently to other respiration-related cues. Furthermore, the role of the depth of respiration warrants further attention. The temperature probe that we used here to measure respiration allows a precise tracking of the time course of the respiratory cycle but does not directly measure respiratory volume, and it could be that varying the depth of respiration while holding the respiratory cycle constant also shapes this co-modulation. In fact, deep breathing forms a cornerstone of typical breathwork, but few studies on the interaction of respiration and cognition have actually measured the depth of respiration or the total airflow.

### (d) Comparison with previous work

A previous study analysed some of the present data using slightly different procedures [28]. While the conclusions drawn previously are supported by the present report, there are a number of technical details that are worthwhile mentioning. First, compared with previous work, we used a revised pre-processing pipeline that assigned a defined respiratory state to a larger fraction of trials, hence losing less of the data. Second, the previous analysis relied on phase-binned data, while in the present study we model the single-trial data against respiration. The latter is particularly relevant when also including additional variables, such as brain activity [1], but also helps avoiding potential biases induced by data binning in general [53,54]. And third, the previous study used both the overall state of respiration (inhalation/exhalation) and the phase within each of these states as separate predictors. While splitting state and phase seems conceptually appropriate given that inhalation and exhalation are physiologically and mechanistically distinct, any downstream influence of these can be time-shifted relative to these by an arbitrary amount and hence this dichotomous interpretation may not be relevant for the interpretation of such effects.

## 5. Conclusion

Making repeated judgements or motor actions comes with trial-to-trial variability in accuracy and speed. Trial-to-trial variations in respiratory phase do explain some of this variability and our results show that this co-modulation follows a systematic time lag. However, respiration is also systematically aligned to expected events, and the phase of alignment is predictive of behaviour across participants. This suggests a profound co-modulation of respiration and perceptual and cognitive performance, which may form the basis for better understanding how conscious respiration and breath-practices can improve brain function or mood, in sports, therapeutic applications or every-day scenarios [55,56].

## Data Availability

Data and scripts are available in the Dryad data repository [57]. Supplementary material is available online [58].
